# Global landscape of patents related to human coronaviruses

**DOI:** 10.7150/ijbs.58807

**Published:** 2021-04-10

**Authors:** Kunmeng Liu, Zixuan Gu, Md Sahidul Islam, Thomas Scherngell, Xiangjun Kong, Jing Zhao, Xin Chen, Yuanjia Hu

**Affiliations:** 1 State Key Laboratory of Quality Research in Chinese Medicine, Institute of Chinese Medical Sciences, University of Macau, Taipa, Macau, China; 2 Innovation Systems & Policy, AIT Austrian Institute of Technology, Vienna, Austria

**Keywords:** coronavirus, COVID-19, patent citation network, patent landscape

## Abstract

At present, the COVID-19 pandemic is running rampant, having caused 2.18 million deaths. Characterizing the global patent landscape of coronaviruses is essential not only for informing research and policy, given the current pandemic crisis, but also for anticipating important future developments. While patents are a promising indicator of technological knowledge production widely used in innovation research, they are often an underused resource in biological sciences. In this study, we present a patent landscape for the seven coronaviruses known to infect humans. The information included in this paper provides a strong intellectual groundwork for the ongoing development of therapeutic agents and vaccines along with a deeper discussion of intellectual property rights under epidemic conditions. The results show that there has been a rapid increase in human coronavirus patents, especially COVID-19 patents. China and the United States play an outstanding role in global cooperation and patent application. The leading role of academic institutions and government is increasingly apparent. Notable technological issues related to human coronaviruses include pharmacochemical treatment, diagnosis of viral infection, viral-vector vaccines, and traditional Chinese medicine. Furthermore, a critical challenge lies in balancing commercial competition, enterprise profit, knowledge sharing, and public interest.

## Introduction

As the Coronavirus Disease 2019 (COVID-19) pandemic unfolds, researchers from all disciplines are coming together and contributing their expertise. At the same time, drug research and design (R&D) and clinical trials are in full swing. The intense development of new therapies is marked by the active use of intellectual property rights along with robust R&D investments 1.

Before 2019, only six coronaviruses—Human Coronavirus 229E (HCoV-229E), Human Coronavirus OC43 (HCoV-OC43), severe acute respiratory syndrome coronavirus (SARS-CoV), Human Coronavirus NL63 (HCoV-NL63), Human Coronavirus HKU1 (HCoV-HKU1), and Middle East respiratory syndrome coronavirus (MERS-CoV)—were known to cause illness in humans 2. The first four are recognized as endemic—that is, these are associated with mild and self-limiting diseases—while the latter two may lead to severe illness and even death. SARS-CoV-2, also called COVID-19, was characterized as a beta coronavirus and is the seventh discrete coronavirus strain that tends to cause severe illness in human beings, which was the reason for a huge, rapidly spreading outbreak of respiratory infections, including potentially fatal pneumonia, that began in Wuhan, China 3.

Given the global impact of the current pandemic situation, a systematic characterization of technological knowledge production is of crucial interest to understanding the global innovation system related to human coronaviruses to inform researchers and policy makers around the world. For characterizing the global landscape of technological knowledge production, innovation research tells us that patents are a highly promising indicator, as they provide systematic information on new technological knowledge disaggregated by very detailed hierarchies of technological fields and attributed in geographical space and time 4. Accordingly, patents might be an important but neglected resource in the biological sciences for characterizing technological knowledge production in specific fields. One ostensible goal of the patent system is to encourage and facilitate the dissemination of technological knowledge. Patents in the life sciences are not only a crucial metric of innovation but also a cornerstone for the commercialization of new fields in life science and healthcare-related technologies. However, there has been very little focus on patent documents as a useful source of information. Further, coronaviruses have commonalities, which are all causing respiratory diseases. In view of the commonness, non-COVID-19 patent documents may be a useful source of information for the R&D of approaches to combating COVID-19^5^.

Here, we present a descriptive study of the coronavirus patent landscape, focusing not only on COVID-19 but on all seven coronaviruses in terms of the common foundation and potential sharing utilization among the different types of coronavirus-based interventions. Previous patent studies have only focused on several subclasses of coronavirus^6^-^9^, such as Choi et al. focused on the MERS-CoV vaccine patents that may have higher potential for commercialization^10^. And no comprehensive patent analyses have been conducted in this area before^5^, ^11^. To fill the gap, we provide an overview of the current trends under development, focusing on the patent landscape of human coronavirus in the temporal, spatial, and technological dimensions, as well as employing a knowledge flow network perspective 12 by using information citations between patents, which may provide a reference for relevant decision making by researchers, investors, and policymakers.

## Materials and Methods

The Derwent Innovation platform (https://clarivate.com/products/derwent-innovation/), a worldwide provider of enhanced patent documents, was used in this study to identify a dataset of patents related to coronaviruses. We searched the titles, abstracts, and claims of patents with a publication date before December 31, 2020, using a series of search terms related to “human coronavirus.” Each patent record consists of detailed information about a Derwent World Patents Index (DWPI) patent family, which includes the basic patent filed in the original country or office and subsequent equivalent patents on the same invention filed in different countries or offices. The patent analysis described below includes time trends, filing countries, patent ownership, co-patents, technological categories, and citation networks and milestones. The items in this patent landscape are reported according to the Reporting Items for Patent Landscapes (RIPL) checklist 13, 14.

The study employs a network analysis perspective to analyze patterns and dynamics of global knowledge flows in the technological domain under consideration. In economics or regional science, network analytical approaches have gained increasing interest in recent investigations of the dynamics of global research and innovation networks 15 as well as knowledge flows 16. The network perspective shifts the emphasis from individual patents to the citation relationships between them. We constructed a series of patent citation networks in which nodes represent patents and directed edges and arrows denote citation relationships and directions. Moreover, patents can be clustered together to form an independent network component, in which nodes have relatively frequent internal connections. The structures and features within patent citation networks comprehensively indicate the patterns of technology flows and evolution. This study also used various descriptive approaches to illustrate the patent indicators and associations between them. The full methodology of the study is described in the supplementary methods section.

## Results

### Data overview

**Figure 1:**
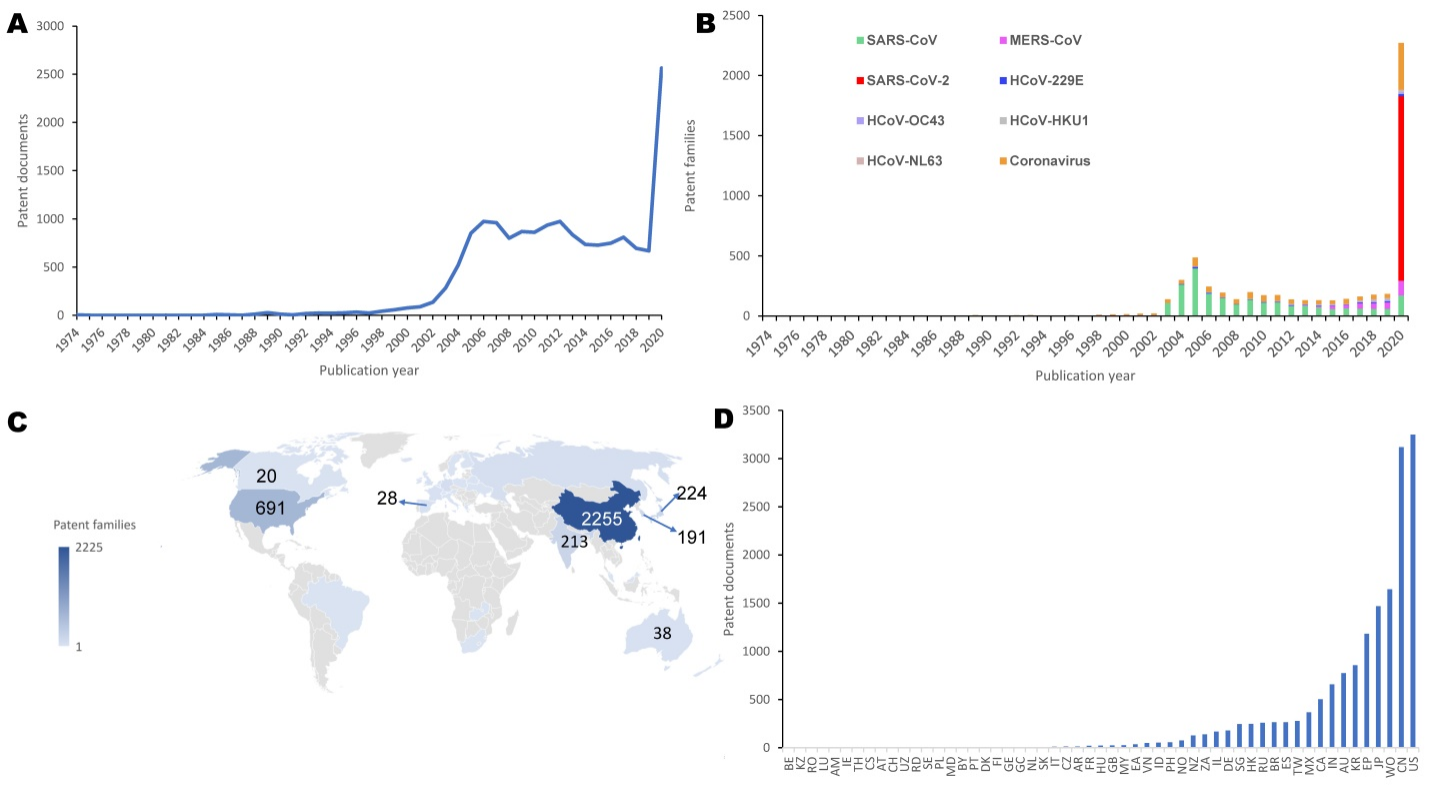
**Temporal and geographic distribution of coronavirus patents.** a. Publication trend (based on patent documents). b. Annual publication change of seven subtypes of human coronavirus and coronavirus type not announced patent (based on patent families). c. Geographic distribution by nationalities of patent inventors. The color intensity denotes the frequency of patent families. d. Geographic distribution by nationalities of jurisdictions (based on patent documents). The "Two-Letter codes" by full country names are shown in Supplementary Table S2.

A dataset of 16,605 patent documents and 5,156 DWPI patent families was generated, including 1,556 patent documents and 1,524 DWPI patent families related to COVID-19. Supplementary information Figure S1 shows the number of excluded and included patents at every stage and the relevant reasons. In 1974, the first coronavirus patent, FR2263769, came into being (the first human coronavirus was discovered in 1964 by Dr. June Almeida) (Figure 1B). In 1988, HCoV-229E and HCoV-OC43 patents (WO8801292; AU8779614) appeared. Patents for HCoV-NL63 and HCoV-HKU1, two other viruses that only cause only mild symptoms, were published in 2005 (EP1508615) and 2006 (US2006018923). Coinciding with the emergence of SARS-CoV, the number of patent documents increased rapidly from 2002 to 2006, plateaued during 2006 to 2019, and peaked in 2020 (Figure 1A). In 2003, SARS-CoV patent CN1439424 appeared, and the number of patent families grew rapidly. MERS-CoV patents appeared in 2012 (e.g., WO2012031090), and their frequency gradually increased until 2019. The number of patent families between 2006 and 2019 was considerably lower than that in 2005, but the number of patent documents remained roughly constant. The first SARS-CoV-2 patent (CN110870402) was added to the human coronavirus patent pool in 2020, and SARS-CoV-2 had gained a large quantity of patent families within 12 months. In 2020, there were more coronavirus patent families than in any other previous year, driven by an explosion in the number of SARS-CoV-2 patents.

### Geographical distribution

We then analyzed the nationalities of patent inventors. We looked at the inventors rather than the applications to trace the locus of knowledge production, a common approach in innovation studies 17. As shown in Figure 1C, countries with the most inventors include China (2,255 patents), the United States (691 patents), Japan (224 patents), India (213 patents), and South Korea (191 patents). This inventor nationality information indicates where the technological source originated. The markets of potential interest can be determined by the patent documents' countries or regions, which included the United States (3,249 patents), China (3,118 patents), Japan (1,469 patents), South Korea (558 patents), and Australia (775 patents), as shown in Figure 1D. The United States, China, Japan, and South Korea were not only the most important markets but also the most productive sources of patents. Beyond these four countries, India has also made considerable efforts in the development of patents related to human coronavirus, while Australia has been an important market.

### Patent assignees

The top 10 assignees by the number of patent families can be seen in Table 1. Most of the top assignees come from China and the United States, indicating that these two countries pay more attention to market protection. Most of the top assignees are from academia and government, from which we identified the Academy of Military Medical Sciences in China as the outstanding player, with 41 inventions filed in 44 patent documents, followed by Fudan University, also in China, and the U.S. Department of Health and Human Services. AstraZeneca, as a representative of the industry, is very active in the field, possessing 35 inventions filed in 476 patent documents. The top assignees among companies possessed a few patent families but many patent documents. By contrast, representative players of academic institutions from China have an obviously small patent family size, which implies that China pays less attention to international patent portfolio strategy.

**Figure 2:**
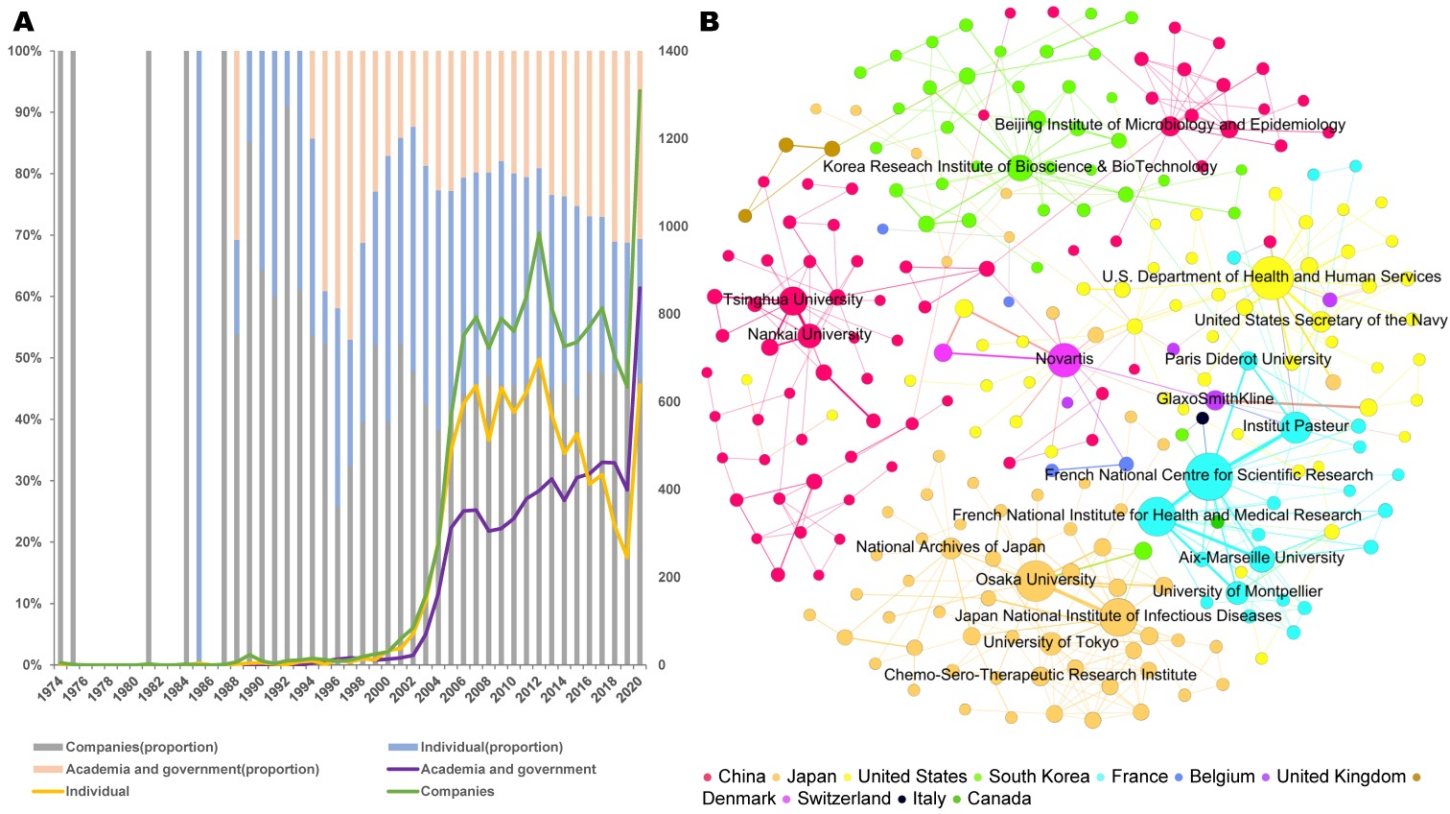
** Types and cooperation of coronavirus patent assignees.** a. Organizational types (based on patent families). b. Collaboration patterns. Institutional collaboration network, in which nodes denote assignees and edges represent co-assignee relations. The main collaboration relationships and patterns among assignees was extracted by network clusters detected using the Louvain modularity method, and labels names of top 20 active institutions. Node size is scaled to the number of patent families, while the thickness of each edge represents collaboration frequency. Countries mean regions in which assignees are located.

Figure 2A shows the change in the organizational types of patent assignees from 1974 to 2020. The proportions of academia and government, industry, or individuals have varied over time. In the early years, patents were only owned by industry players, while academia and government started to own patents in 1988. Since 1993, although industry has still occupied the dominant status, academia and government have played an increasingly important role. The dominant trend of industry shows the mainstream commercialization of human coronavirus-related inventions. In 2016, the number of patents filed by academia and government exceeded that filed by individuals, indicating that the public sector plays an increasingly important role in this field.

The main collaboration relationships and patterns among assignees are shown in the network in Figure 2B, in which there are 258 nodes (assignees) and 453 edges (co-patent ownership relationships among assignees). Among the collaboration patterns, Institut Pasteur and the French National Center for Scientific Research have the closest collaboration. As for the active participants in cooperation, the French National Center for Scientific Research (France), the U.S. Department of Health & Human Services (United States), Osaka University (Japan), the French National Institute of Health and Medical Research (France), the Japan National Institute of Infectious Diseases (Japan), Novartis (Switzerland), Institut Pasteur (France), Tsinghua University (China), the Korea Research Institute of Bioscience and Biotechnology (South Korea), Aix-Marseille University (France), and Nankai University (China) play an important role. Among the top collaborators, academia and government occupy a prominent position, with the U.S. Department of Health and Human Services having the largest number of cooperating partners. China, Japan, the United States, South Korea, and France are actively involved in cooperation. There is considerable collaboration between academia and government, but international collaboration appears to be infrequent, and only three partners of the top 20 collaborators are from industry (Figure 2B). Novartis is the most active company to participate in cooperation. In particular, despite the large number of patents, Chinese organizations rarely collaborate internationally, although they demonstrate extensive domestic cooperation. As a major invention country, the United States actively participates in cooperation with China, Japan, Switzerland, France, and other countries. However, in the human coronavirus field overall, most countries are developing patents independently, and the knowledge boundary between countries is evident.

### Technological characteristics

**Figure 3:**
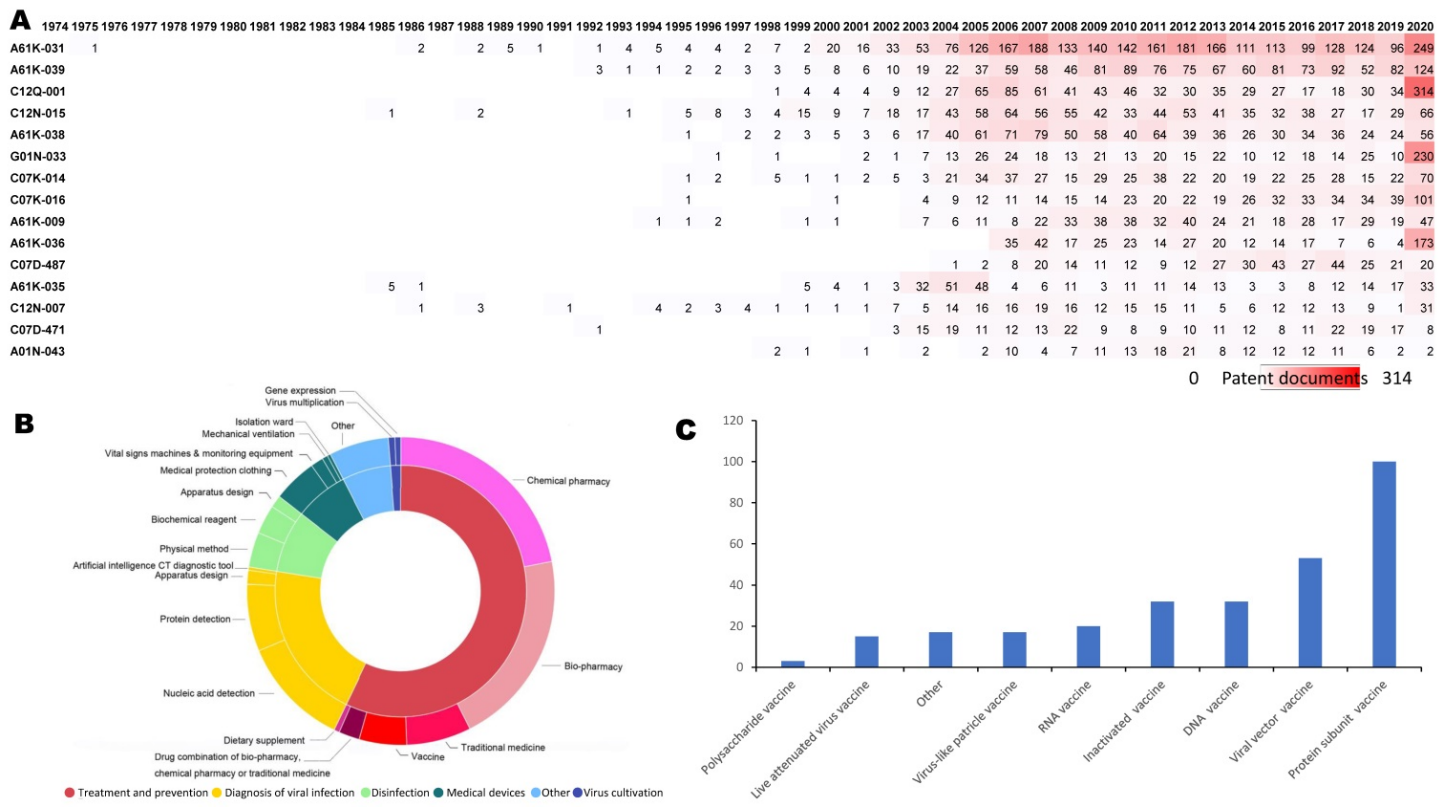
** Technological categories of coronavirus patents.** a. Annual changes of Top 15 International Patent Classification (IPC) codes based on patent families (for further information on IPC codes, visit https://www.wipo.int/classifications/ipc/en/). A61K-031 (Medicinal preparations containing organic active ingredients), A61K-039 (Medicinal preparations containing antigens or antibodies), C12Q-001(Measuring or testing processes involving enzymes, nucleic acids or microorganisms; compositions therefor; processes of preparing such compositions), C12N-015(Mutation or genetic engineering; DNA or RNA concerning genetic engineering, vectors, e.g. plasmids, or their isolation, preparation or purification; use of hosts therefor), A61K-038 (Medicinal preparations containing peptides), G01N-033(Investigating or analysing materials by specific methods), C07K-014(Peptides having more than 20 amino acids; gastrins; somatostatins; melanotropins; derivatives thereof), C07K-016(Immunoglobulins, e.g. monoclonal or polyclonal antibodies), A61K-009(Medicinal preparations characterised by special physical form), A61K-036(Medicinal preparations of undetermined constitution containing material from algae, lichens, fungi or plants, or derivatives thereof, e.g. traditional herbal medicines), C07D-487(Heterocyclic compounds containing nitrogen atoms as the only ring hetero atoms in the condensed system), A61K-035(Medicinal preparations containing materials or reaction products thereof with undetermined constitution), C12N-007(Viruses, e.g. bacteriophages; compositions thereof; preparation or purification thereof), C07D-471(Heterocyclic compounds containing nitrogen atoms as the only ring hetero atoms in the condensed system, at least one ring being a six-membered ring with one nitrogen atom), A01N-043(Biocides, pest repellants or attractants, or plant growth regulators containing heterocyclic compounds). b. Sunburst chart of technological categories (based on patent families). c. Vaccine categories (based on patent families)

To understand how patent applications have changed over time in terms of the direction of technology and investment trends, the annual international patent classification (IPC) trend was analyzed. The assigned IPC categories of the patents indicate the technological areas that the inventions involve. Figure 3A depicts the change in the main IPC categories in seven-digit codes by years. The most prevalent IPC category is A61K-031 (medicinal preparations containing organic active ingredients). In 2003, the quantity of patents for A61K-031 and A61K-035 (medicinal preparations containing materials or reaction products thereof with undetermined constitution) increased suddenly and remained high for the next two years.

In 2012, patents for A61K-031, A61K-039 (medicinal preparations containing antigens or antibodies), C12N-015 (mutation or genetic engineering; DNA or RNA concerning genetic engineering, vectors, e.g., plasmids, or their isolation, preparation or purification; use of hosts therefor) abruptly increased. In 2020, A61K-036 (patents related to detection and traditional herbal medicines), C12Q-001 (measuring or testing processes involving enzymes, nucleic acids, or microorganisms; compositions therefor; processes of preparing such compositions), G01N-033 (investigating or analyzing materials by specific methods), and A61K-031 patents markedly increased. Regarding the proportion of the types of coronavirus, patents related to SARS-CoV accounted for the largest ratio, followed by MERS-CoV, SARS-CoV-2, HCoV-229E, HCoV-OC43, HCoV-HKU1, and HCoV-NL63 (Table 2). The severity of the disease is proportional to the number of patents.

To obtain the technological information related to coronavirus patents, we generated a sunburst diagram representing the patent landscape, which shows six categories and twenty subcategories (Figure 3B). “Treatment and prevention” accounts for the largest proportion (57.89%), followed by “diagnosis of viral infection” (20.64%), “disinfection” (8.12%), “medical devices” (7.00%), and “virus cultivation” (1.32%). In the “treatment and prevention” field, the subcategories “bio-pharmacy” (21.11%) and “chemical pharmacy” (21.51%) occupy the greatest area, and similarities also can be seen in the “vaccine” and “traditional medicine” (5.05% and 6.88%) distribution. Within “diagnosis of viral infection,” “nucleic acid detection” (11.70%) and “protein detection” (7.13%) rank first and second, respectively. Within “disinfection,” the disinfection methods were classified into biochemical reagents (3.11%), physical methods (3.71%), medical devices including medical protection clothing (4.70%), vital signs machines and monitoring equipment (1.34%), mechanical ventilation (0.60%), and isolation wards (0.35%).

To identify the vaccine technologies used, the vaccines were classified into nine groups (Figure 3C). The vaccine type with the highest volume is peptide-based vaccines, with 100 patent families. The rest of the top three vaccine groups are viral-vector vaccines (53 patent families) and DNA vaccines (35 patent families).

### Milestone patents

**Figure 4:**
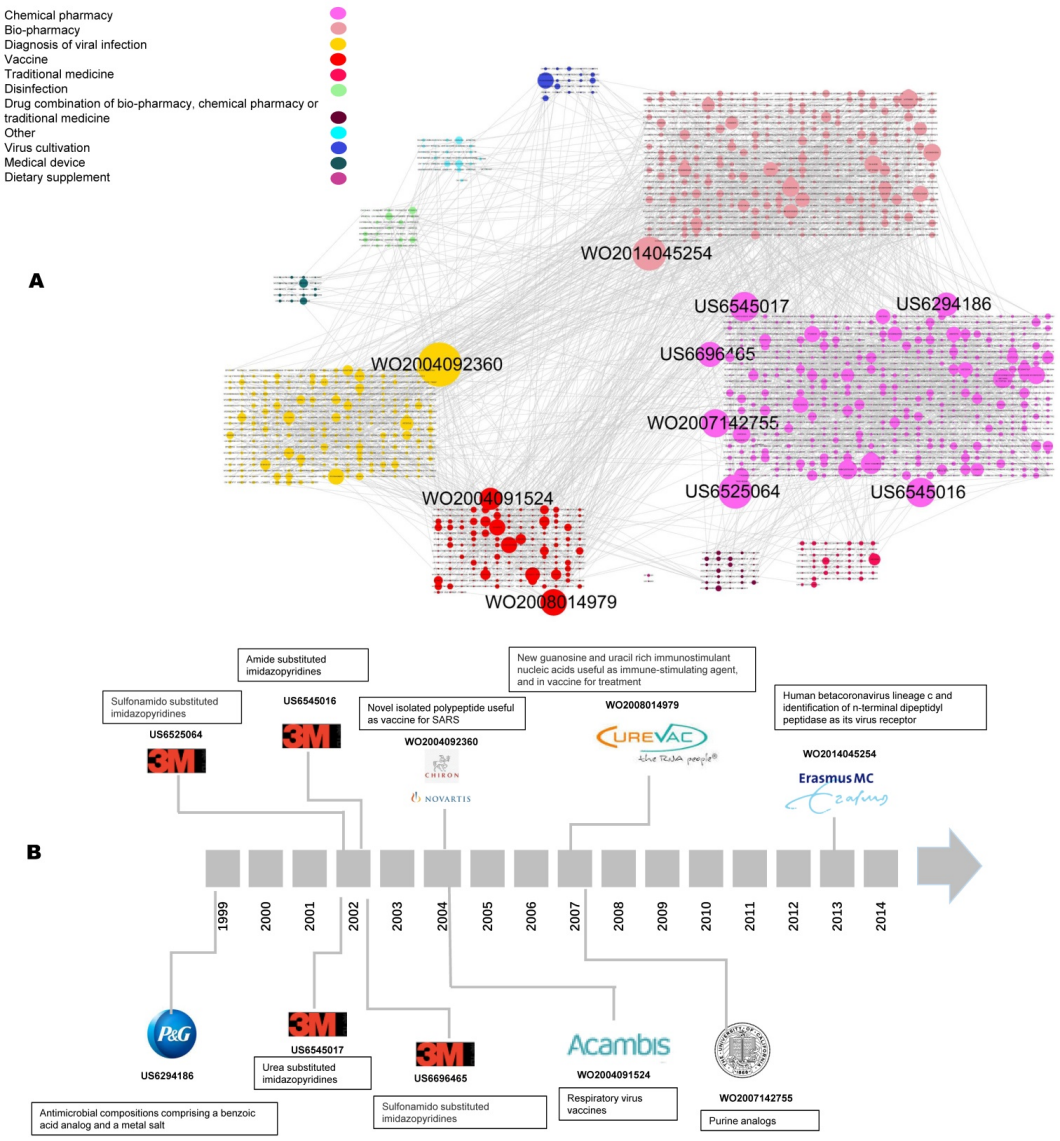
** Citation analysis of coronavirus patents.** a. Global citation network including all patent documents and their citation links. Bigger nodes represent highly cited patents. The node size was set according to its out-degree value, that is, the greater the out-degree, the larger the node size, and the more citations a given patent received (based on technology types). In order to split different technology, the layout was firstly performed by technology classification of the nodes (patents), and then in the same technology module, we use the strategy of “Grid layout” by Cytoscape software. b. The technological temporal route derived from the global patent citation network based on the top 10 citation out-degree.

Figure 4A shows the patent citation networks. In total, 2,149 nodes and 2,675 edges are included. Different colored nodes represent different coronaviruses. Obviously, most of the nodes linked together are of the same color. The more citations of patents, the larger the dot, meaning that more patent assignees imitated this innovation. The yellow community has the biggest node, WO2004092360, which is also shown in the milestones of coronavirus patents (Figure 4B). Most of the top-cited patents are located in the pink clusters. The patent types of “chemical pharmacy,” “bio-pharmacy,” “diagnosis of viral infection,” and “vaccine” form the main part of the citation network.

As for the milestone patents, the route starts with US6294186, applied for in 1999 by Procter & Gamble regarding antimicrobial compositions for coronavirus comprising a benzoic acid analog and a metal salt. The next patents, in 2002, were US6545017, US6525064, US6696465, and US6545016, filed by 3M; these are all pharmaceutical compositions of sulfonamido substituted imidazopyridines for treating the coronavirus. The most-cited patent, WO2004092360, relates to nucleic acids and proteins from SARS and describes the manufacture of diagnostic reagents. WO2004091524, a patent for respiratory virus vaccines from Acambis, is a viral-vector vaccine. WO2007142755 is for purine analogs treating coronavirus, WO2008014979 developed an immune-stimulating agent in the preparation of a vaccine by CureVac, and WO2014045254, applied for in 2013, is about a pharmaceutical composition comprising a human betacoronavirus.

## Discussion

This study identified overall patterns of human coronavirus-relevant patents, including the temporal distribution, geographic scope, organizational assignees, co-patenting activities, and technological focuses among patents. The pattern of patenting activities followed the trend of rapid increases when there was a pandemic of human coronavirus.

### Scientific implications

After the three fatal coronavirus outbreaks, the number of patents increased significantly, and COVID-19 patents showed especially explosive growth. Legal statuses of COVID-19 patents were checked, in which percentage of approved cases is 18.82%. In China, COVID-19 vaccine patents enjoy prioritized examination 18. Many governments have opened special channels for COVID-19 patents “Prioritized Examination”, such as China and the United States, for “Non-Novelty Destroying Disclosure” and “COVID-19 Prioritized Examination Pilot Program”, respectively. The severity of the pandemic, the importance of seizing the market, and the policy of prioritized examination have led to patent outbreaks. After the 2002 SARS outbreak, human coronavirus patents experienced the first round of vigorous growth. During that disaster, more than 5,000 people were infected with SARS coronavirus, including many medical staff, which caused massive panic worldwide 19. The outbreak of the novel coronavirus in late 2019 created the second round of growth and a new peak in patents. A crucial aspect of being prepared for future epidemics is sustained, ongoing research of emerging infectious diseases even during “times of peace" when such viruses do not pose an active threat. In the current situation, the emergency management strategies for COVID-19 consist of blocking transmission, isolation, respiratory and eye protection, and hand hygiene 20, 21. These tactics have led to many related patents. Also, an urgent task is to develop drugs and vaccines. Patents for treatment and prevention received the greatest attention, followed by diagnosis of viral infection, disinfection, and medical devices.

As for vaccines, patents for protein subunit vaccines come in first place, which may result from the fact that they are considered to be significantly safer than whole-pathogen vaccine approaches 22. Peptide-based vaccines account for a large proportion of patents for protein subunit vaccines. Besides the advantages in terms of specificity and safety, the use of peptides as immunogens also offers interesting advantages by lowering vaccine production costs and lead times 23. Patents for viral vector vaccines rank second because the upside of using vectored vaccines is that they are easy and relatively cheap to make 24. Vaccines that have achieved regulatory authorization or approval are EpiVacCorona (Protein Subunit) by FBRI, mRNA-1273 (RNA) by Moderna, BNT162b2 (RNA)by Pfizer/BioNTech, Ad5-nCoV(Viral Vector) by CanSino, Sputnik V (Viral Vector) by Gamaleya, Ad26.COV2.S (Viral Vector) by Janssen (Johnson & Johnson), AZD1222 (Viral Vector) by Oxford/AstraZeneca, Covishield (Viral Vector) by Serum Institute of India, Covaxin (Inactivated) by Bharat Biotech, BBIBP-CorV (Inactivated) by Sinopharm (Beijing), Inactivated (Vero Cells) by Sinopharm (Wuhan), CoronaVac (Inactivated) by Sinovac, CoviVac (Inactivated) by Chumakov Federal Scientific Center for Research and Development of Immune and Biological Products. It can be seen that inactivated vaccines and RNA vaccines, ranking fourth and fifth in the patent landscape, also have good performance. Inactivated vaccines are particularly important in practical terms, as they can be freeze-dried for ease of transportation, which is far more convenient for shipment and storage, a major advantage for both manufacturers and aid groups seeking to transport large quantities of vaccines across the world. The mRNA vaccine by Pfizer-BioNTech and mRNA vaccine by Moderna were the first two approved mRNA vaccine^25^. It is a unique way of making a vaccine, and no such vaccine has been licensed for infectious disease before. Even so, mRNA vaccines could also face supply-chain challenges 26. We need vaccines that are easy to develop and produce, stable for storage and shipping, cost-effective, and safe.

As for medical treatment, on one hand, because of the lack of a specific antiviral medicine, it is urgent to develop vaccines or use antibody treatment. Therefore, patents of medicinal preparations containing antigens or antibodies have been growing continuously. On the other hand, a repurposed drug is the easiest path to take when looking for a new medical treatment with a good understanding of its safety, and the safety evaluation of new drugs takes a long time. The first two launched drugs for COVID-19 are Favipiravir (CN111557939) and Remdesivir (CN111393478, CN111956630, CN111821310, CN111320650, IN2020110226) 27. Favipiravir was originally used to treat Influenza virus infection and Remdesivir for Ebola virus infection.

After the outbreaks of SARS, MERS, and COVID-19, patents classified as A61K-036 increased significantly. Chinese doctors and scientists have suggested using traditional Chinese medicine (TCM) as a source of medicines that purportedly can be used directly against coronaviruses. The reason for this is that TCM has been reported to be effective in the treatment of patients during the COVID-19 pandemic 28. Therefore, the Chinese government has promoted the use of TCM herbs. As a result, 85% of COVID-19 patients in China received combined treatment with regular medication and traditional remedies 29, which led to a large number of related patents.

### Industrial implications

We have shown that scientific and clinical research on coronaviruses is gradually evolving into products of industrial and commercial significance.

Among the top assignees of coronavirus patents, Tsinghua University and Fudan University, both in China, and the University of California in the United States are among the top 100 universities in the world, based on the latest QS World University Rankings, meaning they have the strongest R&D. Dana-Farber Cancer Institute, which did not rank among the top assignees, is also important. Dana-Farber doctor Wayne Marasco and his lab team created the first antibody therapy against SARS in 2004 (WO2007044695, US20050249739). In 2012, they used it to produce the first antibody therapy for MERS-CoV, which spread from Saudi Arabia to a range of other countries. Now Dr. Marasco is working on determining which of the multitude of antibodies in their library represents the best “fit” for the SARS-CoV-2 virus and provides the foundation for antibody-based drugs to treat people with COVID-19 30. Further, AstraZeneca tested the ChAdOx1 nCoV-19 vaccine by cooperating with the University of Oxford 31. The top assignee, the Academy of Military Medical Sciences, shows the military's growing role in medical research in China, and partnerships between China's People's Liberation Army and medical-science companies, such as collaborating with CanSino Biologics to develop the Ad5-nCoV vaccine, have accelerated since the start of the pandemic. In general, non-commercial organizations dominate the list of top assignees. However, inventors from non-commercial organizations are less willing to apply for patents from all over the world to take a position of market share with global patent protection compared with commercial companies.

Moreover, the dominant position of non-commercial organizations is also exhibited in the cooperation patterns. Although industry as the patentee has the largest proportion of patents, academia and government are in first place in the main cooperation patterns. There is a trend of collaboration between public institutions and universities, such as the Japan National Institute of Infectious Diseases and Osaka University, the U.S. Department of Health and Human Services and the University of California, and the French National Center for Scientific Research and Institut Pasteur. University-government research collaboration is important because research funding is mostly sponsored by the government and the government always has the “dominant status” 32. Also, the U.S. Department of Health and Human Services has argued that industry did not fully disclose information on government funding in patent applications relating to vaccines and drug candidates. Public funding matters because it clarifies the right that the government has to ensure that a therapy or vaccine is available to people on reasonable terms. The Moderna mRNA vaccine (US20200282046, US10702600) was awarded a $1.525 billion contract by the Department of Defense and the Department of Health and Human Services to manufacture and deliver 100 million doses of its COVID-19 vaccine 33. Despite this vaccine has been launched, Moderna has been accused of insufficient disclosure because no patents or applications assigned to Moderna disclose United States federal government funding^34^. In the field of infectious diseases, the government expects to have strong control over therapeutic drugs and preventive vaccines to ensure the public can get timely treatment when an epidemic outbreak occurs.

The United States, India, Brazil, and Russia are currently the countries hit the worst by COVID-19. These countries have R&D and economic strength and are actively applying for relevant patents to seize the market. Also, China has been affected by the two fatal infectious coronavirus diseases of SARS and COVID-19 and attaches great importance to combating them. Thus, China is the foremost invention country in this field, and inventors from China have contributed to most of its patents. At the same time, the China National Intellectual Property Administration and the United States Patent and Trademark Office (USPTO) are places where inventors from all over the world seek patent protection. This is because China's pharmaceutical market has been constantly growing in recent years and the U.S. pharmaceutical market is the largest in the world, accounting for more than 40% of global pharmaceuticals sales 35. Also, the United States adopted the “first-to-file principle” after the U.S. Patent Reform Act of 2009, which benefits inventors who are individuals and small businesses 36.

Against the background of the pandemic, the authorization of COVID-19 patents has attracted special attention. The issue of a patent (CN111218459 by Chenwei) makes CanSino's Ad5-nCOV vaccine the first to be approved by China. Almost all drugs are granted patents before they start clinical trials and marketing approval. Because developing a vaccine is such an uphill battle, pharmaceutical companies deserve a patent. A patent helps vaccine makers secure funding for expensive research and additional trials. Pfizer and BioTech developed the BNT162 mRNA-based vaccine candidate (US20200155671) with an immediate $400 million in grants.

Patent competition has begun among countries. China's Wuhan Institute of Virology of the Chinese Academy of Sciences and the Military Medicine Institute of China filed a patent for commercial use in China of Remdesivir, which was invented by Gilead. Gilead has patents for Remdesivir compounds and anti-coronavirus applications (WO2019014247, WO2017049060), namely, basic patents and core patents. Wuhan Institute of Virology also applied for the patent for Remdesivir to treat COVID-19 as a peripheral patent, but the detailed patent information is confidential, before a patent application is published by patent office. Market competition is brutal and fierce. Organizations are employing tactics to maintain their market monopoly. Patents of core knowledge help with earning downstream benefits. Having no promising return revenue will also discourage patent holders 37. But pursuing intellectual property protection for their drugs does not necessarily mean that they intend to aggressively charge monopoly prices. Several covid-19 vaccine providers have indicated that they will provide vaccines at normal prices. Balance is important.

### Social implications

In the early stage of technological development, patent applications do not seem easy to standardize, which is one of the sources of many patent lawsuits. One example is the lawsuits filed by Allele regarding US10221221 and its use in Regeneron's dual-antibody cocktail. Allele accused Regeneron, Pfizer, and BioNTech of infringing patent US10221221, which is for an artificial fluorescent, mNeonGreen, used for testing COVID-19 assays against vaccine candidates. At the same time, the patent infringement lawsuit comes amid escalating China-U.S. tensions, with the Trump administration ramping up efforts to crack down on Chinese enterprises that have become successful in recent years, especially those in the high-tech sector. United States genomics company Illumina won a patent infringement lawsuit against Shenzhen-based BGI Group regarding Modified Nucleotides for Polynucleotide Sequencing (HK1253509A), which is a method used in coronavirus research and involves the next-generation sequencing of genomes. These back-and-forth patent infringement lawsuits between Illumina and BGI are occurring not in a vacuum but in the broader context of escalating Sino-American tensions. Also, CanSino Biologics, one of the leaders in the global push for a coronavirus vaccine, has canceled plans for a clinical trial in Canada after Chinese officials would not let the biotech company ship its product there. These examples demonstrate how international politics may influence and even hinder the response to a pandemic. Amid the epidemic situation, the influence of international politics should be avoided as much as possible.

It is also necessary to provide a public-facing project to ensure universal global access to any COVID-19 treatment and prevention measures. The World Health Organization (WHO) launched a voluntary COVID-19 product pool to collect patent rights, regulatory test data, and other information that could be shared for developing drugs, vaccines, and diagnostics to combat COVID-19 38. One example has been Remdesivir, for which Gilead Sciences has made non-exclusive voluntary licenses for generic pharmaceutical manufacturers without patent royalties, and international trade rules allow nations defined by the United Nations as least-developed countries to ignore patents and make Remdesivir more affordable in their markets 39. The USPTO launched the “Patents 4 Partnerships” program to provide a new patent marketplace for patent owners to voluntarily list patents and published patent applications that are available for licensing in the field of COVID-19 technologies, including its prevention, diagnosis, and treatment. This may be the reason why the United States cooperates more with other countries on patents. At the same time, Oxford University signed an exclusive vaccine deal with AstraZeneca, offering non-exclusive, royalty-free licenses to support free of charge (US9714435, EP2714916), at-cost, or cost-limited margin supply as appropriate, and only for the duration of the pandemic, as defined by the WHO 40. The pandemic has created an extraordinary opportunity for cooperative open-source research, breaking the patent monopoly paradigm 41. To take advantage of this opportunity, all results are encouraged to be shared as quickly as practical and all patents placed in the public domain so that anyone could use them. Massive international effort is a reasonable response to a worldwide crisis in terms of practical challenges like “vaccine nationalism” for low-income countries 42, 43. Faced with global infectious disease threats, major countries are more duty-bound to enhance cooperation between leading institutions or public organizations and industry and to hear the voices of developing countries 44.

All in all, on the one hand, for researchers, emerging infectious diseases should be paid more emphases in advance of epidemics outbreak, especially when viruses do not pose a strong threat. Mining and navigating the state of art from academic papers and inventive patents are of great significance to avoid repeated research work, enhance research effectiveness and efficiency, and impel knowledge spillover and research collaboration. On the other hand, for policymakers, in addition to established accelerating review system on COVID-19 patents, more critical challenges lie in how to narrow down regional inequality obstructing the flow and diffusion of innovative technologies, as well as how to balance innovative motivation and public health demand. The early-stage alert and foresight on key bottleneck technologies from private partners by patent landscaping will be helpful for governments, the public, and industry to run mutual-interest mechanism and to achieve win-win outcome.

One difficulty of uncovering the existing COVID-19 patent landscape is that patent applications are typically not publicly available when filed. Most of the companies developing the current treatment candidates are unlikely to commercialize their products targeting COVID-19 soon. While there are exceptions, patent applications filed with the USPTO are not typically made public for 18 months. Thus, companies may have already filed numerous patent applications earlier 2020 that specifically cover their vaccines, but those applications may not yet be public.

## Conclusion

Since the human coronavirus was discovered, three coronavirus pandemics have occurred. The most recent one, the COVID-19 pandemic, still threatens humans in most areas of the world. In this paper, we reviewed patents concerning seven human coronaviruses and generated a nontraditional technological landscape. Coronavirus patents increased with each pandemic outbreak, with a trend of COVID-19 patents exceeding those related to SARS and MERS. The United States has the largest number of patents, with the majority of top assignees and international cooperation partners. China is also a major inventor country, with an outstanding role in the assignee collaboration network. Though commercially assigned patents form the largest proportion, academia is also an important technological power in global cooperation, and governments themselves hold numerous patent rights. Pharmacochemical treatment, diagnosis of viral infection, and viral-vector vaccines have received great attention, but traditional Chinese medicine is also a research hot spot. Patent infringement, patent conflicts, and disputes over overlapping intellectual property rights should not be ignored. Patent protection is a continuous process under commercial competition, especially in a global patent portfolio strategy. In epidemics, the role of patent rights is different from that under normal circumstances, and more attention should be paid to balancing competing interests and the humanistic spirit with the general trend of knowledge sharing.

## Supplementary Material

Supplementary method, figure and tables.

## Figures and Tables

**Table 1:** Top assignees of coronavirus patents

Rank	Assignees	Patent documents	Patent families	Average number of patents per family	Assignee type
1	Academy of Military Medical Sciences (China)	44	41	1.07	A&G
2	Fudan University (China)	43	38	1.13	A&G
3	U.S. Department of Health and Human Services (US)	176	37	4.76	A&G
4	AstraZeneca (Sweden)	476	35	13.60	C
5	Tsinghua University (China)	54	30	1.80	A&G
6	University of California (US)	137	29	4.72	A&G
7	Korea Research Institute of Bioscience and Biotechnology (South Korea)	104	25	4.16	A&G
8	Novartis (Switzerland)	323	23	14.04	C
9	3M Company (US)	220	23	9.57	C
10	InterMune (US)	165	21	7.86	C

Abbreviations: C: company; A&G: academia and government. Average number of patents per family= the number of patent documents / the number of patent families.

**Table 2:** The number of patent documents and patent families for each type of coronavirus

Coronavirus type	Number of patent families	Number of patent documents
SARS-CoV	2275	9153
MERS-CoV	333	887
SARS-CoV-2	1524	1556
HCoV-229E	109	402
HCoV-OC43	87	301
HCoV-HKU1	46	251
HCoV-NL63	75	121
Coronavirus type not announced	1206	5444

## Abbreviations

SARS: severe acute respiratory syndrome
SARS-CoV: severe acute respiratory syndrome coronavirus
MERS: Middle East respiratory syndrome
MERS-Cov: Middle east respiratory syndrome coronavirus
HCoV-229E: human coronavirus 229E
HCoV-OC43: human coronavirus OC43
HCoV-NL63: human coronavirus NL63
HCoV-HKU1: human coronavirus HKU1
COVID-19: coronavirus disease 2019
SARS-CoV-2: severe acute respiratory syndrome coronavirus 2
TCM: traditional chinese medicine
DWPI: derwent world patents index
USPTO: United States patent and trademark office
IPC: international patent classification
