# Supplementary material for: Global landscape of patents related to human coronaviruses
Source: Int J Biol Sci. 2021 Apr 10;17(6):1588–99. doi: 10.7150/ijbs.58807 (PMC8071764; doi:10.7150/ijbs.58807)
Supplement: Supplementary file 1 — Supplementary method, figure and tables. [file ijbsv17p1588s1.pdf]

Supplementary Materials

1. Supplementary method

1.1 Patent search

To search specifically for Human Coronavirus patents, the Derwent Innovation® (Clarivate Analytics®) platform (https://clarivate.com/products/derwent-innovation/), a comprehensive database which include globally patent data was used. A search query was developed to retrieve patents related to human coronavirus invention, which was performed based on keywords.

As is common in a patent landscaping activity, one of the first steps is the definition of a search strategy in order to retrieve the most relevant set of documents. These documents, at a later stage, will form the data set to be analysed. Keyword searches were performed in the title, abstract and claims sections of patent documents. Keywords included the full names of the seven coronaviruses, abbreviations, or synonyms.

In all, the following search queries were used: Topic= “MERS-CoV” OR “SARS-CoV” OR “SARS-CoV-2” OR “COVID*" OR “2019-nCoV” OR “2019 nCoV” OR “CoV-229E” OR ”CoV-OC43” OR ”CoV-NL63” OR ”CoV-HKU1” OR ”HCoV-229E” OR ”HCoV-OC43” OR ”HCoV-NL63” OR “HCoV-HKU1” OR “corona virus*” OR “coronavirus*” OR “severe acute respiratory syndrome” OR “Middle East respiratory syndrome” OR “sars” OR “mers”

1.2 Timeframe and analysed documents

All searches were performed for the period 1963 to 2020 of patent publication year. For counting inventions, the simple patent family was used in the study. A simple patent family was defined by Derwent World Patent Index (DWPI) family criteria as a collection of patent documents that are considered to cover a single invention. The technical content covered by the applications is considered to be identical. Members of a DWPI patent family will all have exactly the same priorities[1]. Besides we excluded irrelevant patents by double-checking manually, and then deduplicate records, e.g., different document types (e.g. A1, B2, C) of a given publication to avoid multiple counts for the same invention. As for the dataset cleaning, a fundamental data normalization step was performed to clean duplicate records that had occurred due to abbreviations or misspelling. The unrelated patents were deleted in the document records after being double-checked manually (Figure S1). We conducted data

cleaning automatically and manually with reference to the Preferred Reporting Items for Systematic Reviews and Meta-Analyses (PRISMA)[2, 3], and the items in this patent landscape are reported according to the Reporting Items for Patent Landscapes (RIPL) checklist[4, 5]. Here, after reading the title, abstract, claim and full text of patent files, we deleted all the patents related to the coronavirus of animals and only keep the patents of human coronavirus. This study did not exclude patent applications that are still pending, in order to capture the latest progress in patenting activities due to rapid developments in recent years. Patents are the dissemination and exchange of information, even if a patent is not approved. Therefore, published patents including pending ones are all necessary for our research, in terms of the patent is the text of a technology. The search was performed on 2020 December 31th and a total of 16605 records (patent documents) were found with 5156 Derwent World Patent Index (DWPI) families (patent families).

1.3 Classification of patents

We collected the information about the classification of patents of technological characteristics based on previous research[6-8] in order to capture technological features of the patent files. According to the previous literature and the actual situation of patent application, the overall patents were classified into six general categories and twenty subcategories. The patents of vaccines were divided into nine categories. We further marked classification labels by a hierarchical reading order from the title, abstract, claims, and full-text. Table S1 shows more details.

1.4 Collaboration network among co-assignees

In the collaboration network, nodes denote assignees and edges represent co-assignee relations. If there is an edge between two nodes, it means the two assignees jointly hold one patent family and there is a cooperation relation. In a collaboration network among co-assignees, we followed full counting procedure, wherein a patent family owned by n assignees produces n(n-1)/2 links (edges) that we count. For instance, for a patent family with three assignees, we counted three links (edges): from assignee a to assignee b, from b to c and from a to c. The size of the node represents the patent family count, and the thickness of the edge indicates co-assignees’ count. The thicker the edges, the more cooperation. All nodes are colored based on the country of assignees. The nodes were distributed using the “Fruchterman Reingold”[9] following manual adjustment, which is a force-directed layout algorithm. The Gephi software provides a community detection algorithm by Louvain method[10].

The clusters with more than 15 nodes were thus extracted by pressing “Modularity” in Gephi to generate isolated largest cooperative group.

1.5 Patent citation network and technology route

We created a patent citation network to uncover the clusters of technological trends in human coronavirus inventions based on knowledge flows by mapping the main path of patent citation. The citation one was created using information from the “Publication number” (source) and “Cited patents” (target) columns from the original dataset. The dataset containing the link between the citing-cited patent were imported into Cytoscape[11] to implement our data visualization. The network of patent citation is a directed graph, which represents the link from the citing patent to the cited patent. The global network of patent citation related to Human Coronavirus is designed by 2149 nodes and 2675 edges. The nodes are colored based on different technology and their size was set according to their out-degree value.

A module (cluster) in the citation network is a group of nodes with tightly connected edges with each other and the nodes of module show similar characteristics. Module represents a high degree of internal connection and a close relationship between nodes in the social network analysis. According to our research needs, we paid more attention to the important patents in different technology categories. Therefore, we put the nodes of same category of technology or function close to each other to form the modules. In order to split different technology, the layout was firstly performed by technology classification of the nodes (patents), and then in the same technology module, we use the strategy of “Grid layout” by Cytoscape software. All patents that were labeled with their own publication number in Figure 4A can be seen by zoom.

We identified patents that may represent major innovations via patent citation analysis. According to the “priority year”, the most cited patents of top ten is regarded as the milestone patents to form the technology route, and these patents are shown in the timeline. The patent codes and the English titles are displayed in the text, and the assignees’ information is displayed in the form of a logo.

References

1. Clarivate. Derwent innovation: Dwpi and inpadoc family criteria. 2020.

2. Hutton B, Salanti G, Caldwell DM, et al. The prisma extension statement for reporting of systematic reviews incorporating network meta-analyses of health care interventions: Checklist and explanations. Annals of Internal Medicine. 2015; 162: 777-84.
3. Moher D, Liberati A, Tetzlaff J, et al. Preferred reporting items for systematic reviews and meta-analyses: The prisma statement. International Journal of Surgery. 2010; 8: 336-41.
4. Smith JA, Arshad Z, Trippe A, et al. The reporting items for patent landscapes statement. Nature biotechnology. 2018; 36: 1043-8.
5. Smith JA. Improving transparency and reproducibility of patent landscapes: The reporting items for patent landscapes (ripl) statement and other considerations. World Patent Information. 2020; 62: 3.
6. Callaway E. The race for coronavirus vaccines: A graphical guide. Nature. 2020; 580: 576.
7. Le TT, Andreadakis Z, Kumar A, et al. The covid-19 vaccine development landscape. Nature Reviews Drug Discovery. 2020; 19: 305-6.
8. Dai L, Gao GF. Viral targets for vaccines against covid-19. Nature Reviews Immunology. 2020: 1-10.
9. Fruchterman TMJ, Reingold EM. Graph drawing by force-directed placement. 1991; 21: 1129-64.
10. Blondel VD, Guillaume J-L, Lambiotte R, et al. Fast unfolding of communities in large networks. Journal of Statistical Mechanics: Theory and Experiment. 2008; 2008.
11. Shannon P. Cytoscape: A software environment for integrated models of biomolecular interaction networks. Genome Research. 2003; 13: 2498-504.

2. Supplementary figure

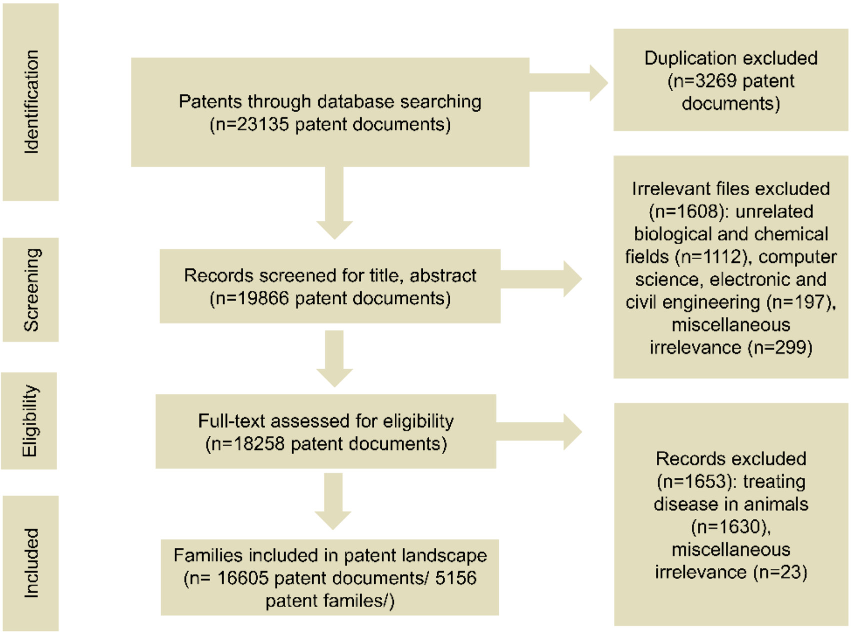

Figure S1. PRISMA flow diagram detailing the number of patent documents included at each stage and the reasons for removal.

3. Supplementary table

Table S1. The standardization of patent classification.

**Case**	**Classification**
Title	If the classification information is clearly shown in the title of a patent document, we labelled the patent by the classification. For example, patent CN111375055 is entitled “Vaccine composition useful against novel coronavirus (2019-nCoV) and HIV, comprises virus envelope subunit vaccine and glucopyranosyl lipid adjuvant”. We chose the “Vaccine” as the classification.
Abstract	If there is no classification information mentioned in the title, we further captured the classification information in the abstract. For example, the abstract of patent CN111647055 is “The invention belongs to the technical field of immunization, and particularly relates to a recombinant antigen for novel coronavirus detection and preparation and application thereof.” We chose the ”Diagnosis of viral infection“ as the classification.
Claims	If there is no classification information both in title and in abstract, we continued to check patent claims. In most cases, by reading the title and abstract, we can divide the patents into general categories. If we can't determine the subcategory by reading the title and summary, we need to refer to patents claims to classify the subcategories under the general category. Make decisions based on the level of priority claim. For example, patent US2020281972 claims that “Claim 1. A method of treating at least one condition caused by a coronavirus or an influenza virus, the method comprising administering a composition comprising copper ions to a subject in need thereof.” We recognized that the patent covers "Chemical pharmacy" under “treatment and prevention”.
Full-text	When none of the above ways can determine the category, we will read the full text.

Table S2. Country codes of patents by jurisdiction.

**Abbreviation**	**Full name**
BE	Belgium
KZ	Kazakhstan
RO	Romania
LU	Luxembourg
AM	Armenia
IE	Ireland
TH	Thailand
CS	Czechoslovakia (up to 1993)
AT	Austria

CH	Switzerland
UZ	Uzbekistan
RD	#N/A
SE	Sweden
PL	Poland
MD	Republic of Moldova
BY	Belarus
PT	Portugal
DK	Denmark
FI	Finland
GE	Georgia
GC	Golf Cooperation Council
NL	Netherlands (the)
SK	Slovakia
IT	Italy
CZ	Czech Republic
AR	Argentina
FR	France
HU	Hungary
GB	United Kingdom (the)
MY	Malaysia
EA	Eurasian Patent Organisation
VN	Viet Nam
ID	Indonesia
PH	Philippines (the)
NO	Norway
NZ	New Zealand
ZA	South Africa
IL	Israel
DE	Germany
SG	Singapore
HK	Hong Kong (China)
RU	Russian Federation
BR	Brazil
ES	Spain
TW	Chinese Taipei
MX	Mexico
CA	Canada
IN	India
AU	Australia
KR	Republic of Korea
EP	European Patent Office
JP	Japan
WO	World Intellectual Property Organization
CN	China
US	United States of America
